# Visualization of Biosurfactant Film Flow in a *Bacillus subtilis* Swarm Colony on an Agar Plate

**DOI:** 10.3390/ijms160920225

**Published:** 2015-08-26

**Authors:** Kyunghoon Kim, Jung Kyung Kim

**Affiliations:** 1 Department of Mechanical and Aerospace Engineering, North Carolina State University, Raleigh, NC 27695, USA; E-Mail: kkim15@ncsu.edu; 2 Department of Mechanical Engineering, Kookmin University, Seoul 02707, Korea; 3 Department of Integrative Biomedical Science and Engineering, Kookmin University, Seoul 02707, Korea

**Keywords:** bacterial swarming, *Bacillus subtilis*, swarm colony, flow, fluorescent microbeads, particle image velocimetry

## Abstract

Collective bacterial dynamics plays a crucial role in colony development. Although many research groups have studied the behavior of fluidic swarm colonies, the detailed mechanics of its motion remains elusive. Here, we developed a visualization method using submicron fluorescent beads for investigating the flow field in a thin layer of fluid that covers a *Bacillus subtilis* swarm colony growing on an agar plate. The beads were initially embedded in the agar plate and subsequently distributed spontaneously at the upper surface of the expanding colony. We conducted long-term live cell imaging of the *B. subtilis* colony using the fluorescent tracers, and obtained high-resolution velocity maps of microscale vortices in the swarm colony using particle image velocimetry. A distinct periodic fluctuation in the average speed and vorticity of flow in swarm colony was observed at the inner region of the colony, and correlated with the switch between bacterial swarming and growth phases. At the advancing edge of the colony, both the magnitudes of velocity and vorticity of flow in swarm colony were inversely correlated with the spreading speed of the swarm edge. The advanced imaging tool developed in this study would facilitate further understanding of the effect of micro vortices in swarm colony on the collective dynamics of bacteria.

## 1. Introduction

Biosurfactants secreted via the quorum sensing mechanism play important roles in biofilm and swarm dynamics [1,2]. The fluidic properties of biosurfactant film flow in a swarm colony vary according to the type of biosurfactant. Bacterial cells in swarm colony have collective behaviors such as swarming, which is powered by rotating helical flagella. Swarming is the collective motion of bacterial cells across a surface, and it requires a significant amount of energy, a solid surface, and other specific conditions. *Bacillus subtilis* cells swarm on a wide range of energy-rich media and require soft agar in a narrow range of concentrations. Agar concentrations above 0.3% (*w*/*v*) are favorable for promoting swarming, whereas agar concentrations above 1% prohibit swarming of many bacterial species. Therefore, the 1.5% standard agar plate concentration used in the laboratory can be considered a substrate that inhibits swarming [3].

Understanding the mechanical properties and fluidic characteristics of a bacterial colony can enhance studies on a bacteria colony. Tuson *et al.* [4] developed a technique for measuring the mechanical properties of bacteria *in vivo*. They considered the complex structure of the cell wall and used stiffness as an effective Young’s modulus. This resulted in a model of the bacterial cell wall of specific bacteria in limited ranges, which was designed using mechanical concepts such as the rigid network model and stiffness. Shaw *et al.* [5] demonstrated that biofilm structure in bacterial colonies is not just a simple material but is also complex with fluidic characteristics such as viscoelasticity. This concept provides useful tools for studying the biophysics of bacteria.

The dynamics of various swarm colonies have been investigated using small tracer particles. Zhang *et al.* [6] used magnesium oxide (MgO) smoke particles deposited on the top surface of an *Escherichia coli* swarm to confirm the existence of the stationary interface between the swarm and air. In contrast, Be’er and Harshey [7] observed super-diffusivity of MgO particles on the upper surface of biosurfactant-producing swarms of *Serratia marcescens* and *B. subtilis* using a single particle tracking method. In our previous studies, we measured the velocity of bacterial cells grown on agar plates of different agar concentrations between 0.5% and 2.5% using a multi-particle tracking technique, and found that bacteria in the swarm colony have the ability of periodic switching between the growth and swarming phases [8,9].

Although several studies have investigated the behavior of a fluidic swarm colony, the exact mechanism of their motion is still poorly understood. To the best of our knowledge, there are no reports in the literature concerning the observation of a detailed flow field in a swarming bacterial colony. In the present study, a high-resolution particle image velocimetry (PIV) for visualizing the flow pattern in a swarm colony of *B. subtilis* was developed using submicron fluorescent particles. We studied an interplay between the spreading rate of the *B. subtilis* swarm edge and microscale fluid dynamics at the upper surface of the swarm through long-term time-lapse imaging of advancing swarm edge and the submicron fluorescent beads that were initially embedded in the agar plate and distributed spontaneously on the upper surface of the growing colony.

## 2. Results and Discussion

### 2.1. Fluorescent Bead Dynamics Influenced by Swarming Bacterial Cells

**Figure 1:**
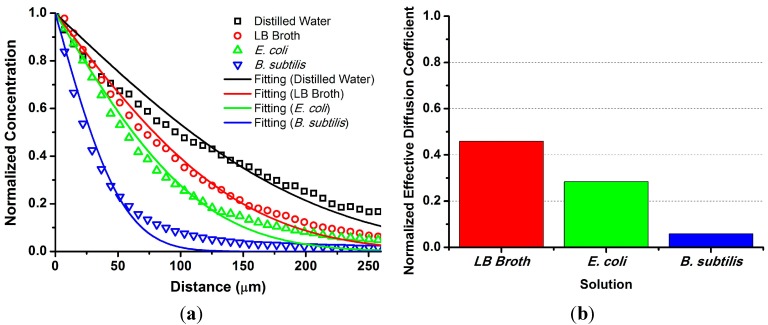
Dynamics of the submicron fluorescent beads. (**a**) Fluorescence intensity profile of the beads in each solution at 30 s after initiation of diffusion from the 0.5% agar plate. The symbols and lines represent experimental data and regression curves obtained by Equation (1), respectively; and (**b**) Comparison of *D*_eff_ of the bead in different solutions. Measured *D*_eff_ of the beads in LB Broth (LB), supernatants of *E. coli* and *B. subtilis* were normalized by *D*_eff_ in distilled water (DW). The relative magnitudes are DW > LB > *E. coli* > *B. subtilis*.

Escaping of the fluorescent beads embedded in the top layer of the agar plate was caused by diffusion across the concentration gradient between the solution and the agar gel. Both the fluorescence intensity profile of the bead distribution in each solution and the regression curve produced by the Equation (1) at 30 s after initiation of diffusion are plotted in Figure 1a. The regression curves are well matched with the variation trends of measured data. We estimated an effective diffusion coefficient (*D*_eff_) of the bead by the regression analysis and compared *D*_eff_ in different solutions such as distilled water, Luria-Bertani (LB) broth, and supernatants of *E. coli* and *B. subtilis*. *D*_eff_ was highest in the distilled water and the magnitude was decreased in the order of LB Broth, *E. coli*, and *B. subtilis* as shown in Figure 1b. We demonstrated that the movement of the fluorescent beads from the agar gel was induced by diffusion mechanism. In the regression analysis, the coefficient of determination (*R*^2^) was higher than 0.95 and the average residual was lower than 0.05. When the surface of the agar plate was more dried, more beads were escaped from the agar gel. This result is consistent with a previous finding that the spatial gradient of the biosurfactant concentration leads to marangoni effect [10], and provides a clue to our observation that the fluorescent beads in the *B. subtilis* colony secreting biosurfactant get away from the agar plate, and the beads in the *E. coli* colony secreting wetting agent are immobilized at the upper surface of the colony [11].

Based on the aforementioned findings, we can propose a model for fluorescent bead dynamics affected by swarming bacterial cells. When a droplet of bacterial suspension is initially inoculated into the surface of the bead-coated agar plate, the beads in the agar gel leak into the swarm colony by diffusion. While the swarm colony grows gradually, the beads are distributed on the upper surface of the colony. Eventually, when a group of bacterial cells secretes the biosurfactant and starts swarming in the colony, the motion of the beads are driven by biosurfactant flow which is influenced by physicochemical fluid properties such as viscosity, wettability, and surface tension.

Steager *et al.* [12] observed experimentally that the bacteria featured the combination of curvilinear translation and vortex modes, and Lushi *et al.* [13] reported a mathematical modeling of collective motion in bacterial cells. Lega and Passot [14] also proposed a mathematical model for hydrodynamics of bacterial colonies by regarding the bacterial colony as a two-phase fluid composed of a fluid phase and a particulate phase, but they did not investigate the marangoni effect in the colonies. These studies indicate that a number of researchers has considered flow in the colonies, and it is reasonable to assume that the swarm colony is a complex biofluid system. Furthermore, as Be’er and Harshey [7] mentioned that superdiffusive behavior is caused by the interaction of the collective motion of the bacteria within swarm, the bacterial cells in the *B. subtilis* colony initially touch the beads or make flow stream generating shear stress and consequently the movement of the beads is driven by the adjacent viscous flow. Putting these things together, we suggest that the motion of the beads are influenced by flow as well as bacterial cells.

### 2.2. Mean Flow Velocity and Swarming Dynamics

**Figure 2:**
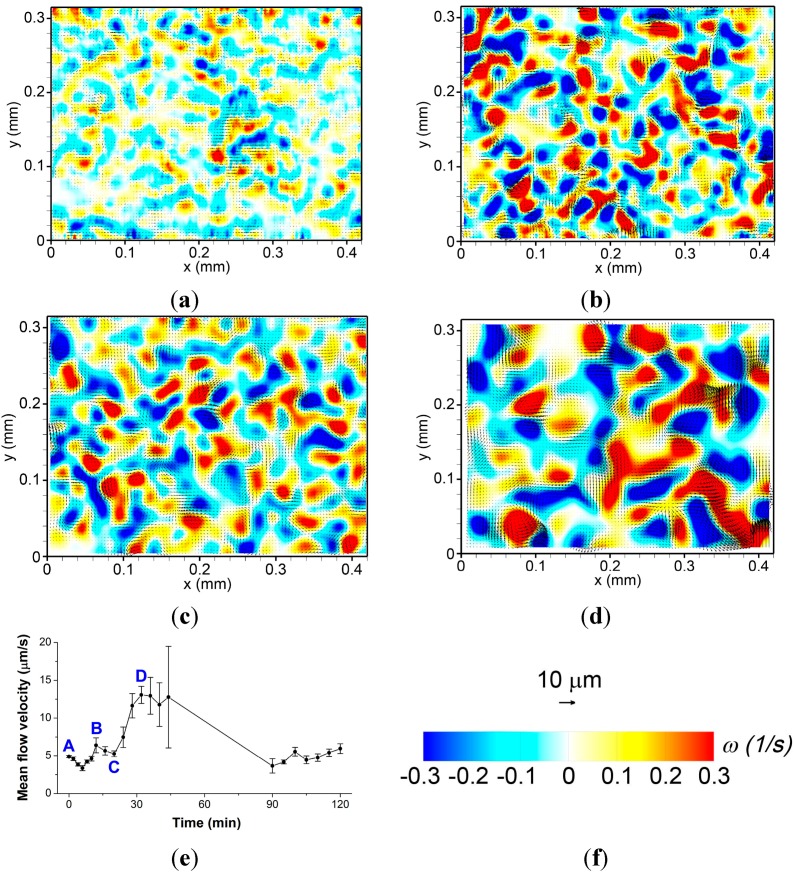
Instantaneous velocity (arrow) and vorticity (color) maps of swarm colony flow at the interior of the *B. subtilis* colony obtained by particle image velocimetry at (**a**) 0; (**b**) 12; (**c**) 20; and (**d**) 32 min after initiation of swarm motility; (**e**) Variation in the mean flow velocity with respect to time; and (**f**) Scales of the velocity vector and vorticity. Colors indicate the strength of relative vorticity, red for positive (counter-clockwise rotation) and blue for negative (clockwise rotation) vorticity, respectively.

The average speed of swarm colony flow for 2 h was displayed in Figure 2e. We used a cut-off time of 90 min because the cyclic variation of the mean flow velocity with a period of 90 min was observed. There was a blank period from 56 to 90 min because erroneous flow velocity vectors were processed in that period due to poor quality of the fluorescent bead images. The mean flow velocity fluctuated initially within 8 min and then increased gradually to yield an averaged value of 6.7 µm/s.

In our prior study, we measured the velocity of moving bacterial cells grown on an agar plate and found that bacterial velocity would change periodically in association with their growth and swarming phases [8]. However, it was not confirmed whether this periodic change in velocity was a common or rare phenomenon. Here we measured the average velocity of swarm colony flow induced by bacterial swarming for long term with higher temporal resolution and observed that their regular velocity patterns varied. If the motion of the bacterial cell displays regular patterns, then the bacterial cell would secrete a self-produced matrix of extracellular polymeric substance that serves as microenvironment for the bacteria. Therefore, swarm colony flow is influenced by the secretion of the bacterial cell and this may affect the regularity of the velocity pattern. The fluctuation in the average velocity of the swarm colony after long periods can be attributed to the regularity of swarm colony flow reflected in the bacterial cell. The average velocity of bacterial cells observed by Yoon *et al.* [8] was approximately 10 µm/s in the swarming phase, and the average velocity measured by Phuyal and Kim [15] was higher than 20 µm/s. In the present study, the average velocity of the swarm colony flow was 6.7 µm/s. These findings are reasonable since viscous swarm colony flow generated by movements of the bacterial cells should be slower than the bacterial cell.

A group of bacterial cells grown on an agar plate undergoes multiple phases, including a consolidation phase involving growth and a swarming phase associated with collective behavior. Bacterial behavior is impacted by their phase [16]. When bacteria are in the growth phase, they exhibit low activities but when they are close to the swarming phase, bacterial motion gradually ascends until the swarming phase ends. Subsequently, this trend towards increasing activity weakens since they are close to the growth and multiplying phases. Since this process coincides with swarm colony flow dynamics, it is important to assess the regularity of velocity patterns. We previously reported that *D*_eff_ of 200 nm particle in a *B. subtilis* suspension was 33.83 µm^2^/s [9]. Wu and Berg [17] determined that *D*_eff_ of 2 µm microbubble was 31 ± 1 and 64 ± 2 µm^2^/s in the monolayer and the multilayer of a *B. subtilis* colony, respectively. Bacterial swarming would affect the magnitude of *D*_eff_ and the viscosity of the fluidic swarm colony as well. These findings support our data since the average flow velocity changed regularly with respect to time. The bacterial cells in a colony multiply, increase in thickness, and become multilayered over time. Therefore, the overall flow velocity has increased gradually in the swarming phase.

### 2.3. Velocity and Vorticity Distributions at the Interior of the Colony

We first observed the layer of bacterial cells and identified its depthwise position. Focusing on the bacterial cells in the bright-field mode, we switched to the fluorescence mode to change the focus onto the fluorescent beads. The fluorescent beads were found to be distributed approximately 15 μm above the cells and proved to be appropriate tracers for the swarm colony flow. The dynamics of several swarm colony have been observed and analyzed by single particle tracking method [6,7]. However, the observation and analysis of swarm colony flow lacked sufficient spatial resolution for studying fluid dynamics in detail.

The *E. coli* surfactant is rich wetting and has strong surface tension; however, that of *B. subtilis* is poor wetting and has weak surface tension [18]. In this study, we utilized fluorescent particles, which provide the advantage of exploiting differences in the surface tension, according to the characteristic of the swarm colony, since fluorescent particles were distributed evenly around the flow field. Figure 2 shows the instantaneous velocity vector distribution (arrow) and vorticity (color) map of the swarm colony flow inside the *B. subtilis* colony at four distinct time points denoted in Figure 2e, which plots the variation of the mean flow velocity with respect to time. Scales of the velocity vector and vorticity are shown in Figure 2f, where colors indicate the strength of relative vorticity, red for positive (counter-clockwise rotation) and blue for negative (clockwise rotation) vorticity, respectively. Figure 2a shows the movement of the beads with the initiation of swarming, which is set as *t* = 0, approximately 1 s after swarming begins. Figure 2b,d represent the high-velocity distribution at *t* = 12 min and *t* = 32 min, respectively. Figure 2c represents the low-velocity distribution at *t* = 20 min.

Wilking *et al.* [19] demonstrated that the direction of fluid flow is inward at the edge of the colony, due to the concentration gradient of extracellular matrix polymer. We therefore hypothesize that when the average velocity of the swarm colony flow is low, the polymer concentration gradient is dominant. However, when the average flow velocity is high, the bacterial behavior is reflected in the entire area of the colony. In addition, the swarm colony flow was influenced more by the polymer concentration gradient than by the movement of bacterial cells at the initial phase. In general, weak vortices were distributed non-uniformly at the initial phase. Therefore, we conclude that low-average velocity was affected by the weak velocity vector magnitude, and high-average velocity was affected by the strong velocity vector magnitude. We further conclude that the velocity vector influences the magnitude of vorticity. We analyzed complex vorticity maps over time. As demonstrated with the colored map of the vorticity distribution in Figure 2, the flow vorticity was irregular and unsteady. The influence of the cooperative behavior of bacterial cells affects swarm colony flow vorticity to an extent. It is known that strong velocity gradient induces intensified vorticity. However, the reason why bacterial colonies have irregular and complex behavior is not clear yet. In order to address this issue, it is necessary to measure the viscosity of the swarming phase *in situ* and compare the viscous property in the swarming phase to that in other phases.

### 2.4. Flow Near the Advancing Edge of the Swarm

**Figure 3:**
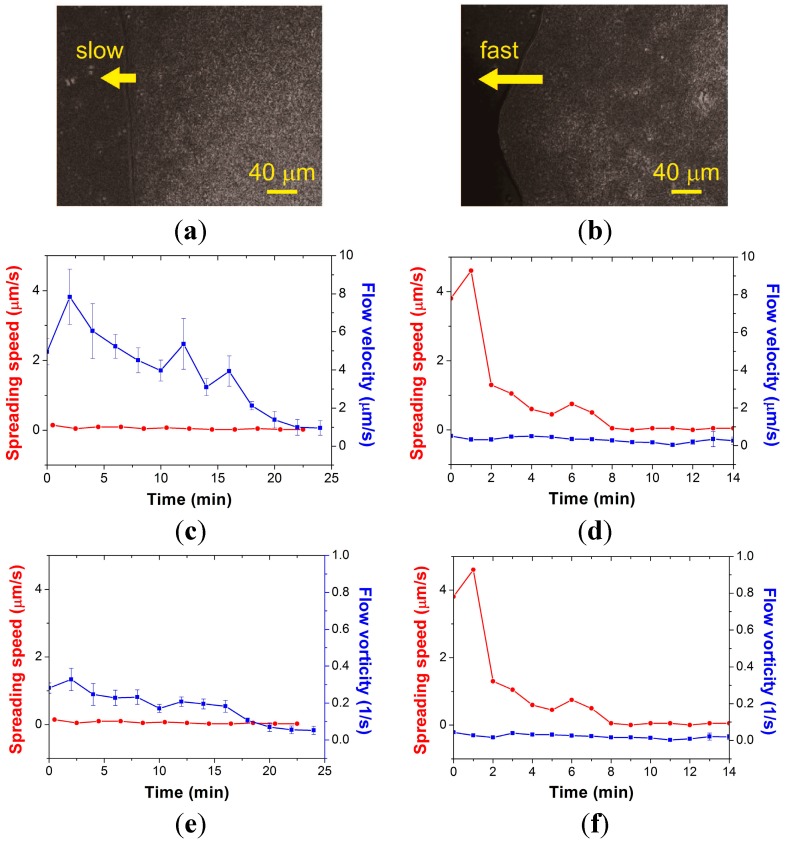
Bright-field image near the advancing edge of the *B. subtilis* colony in (**a**) slow spreading and (**b**) fast spreading mode. The average flow velocity and vorticity were calculated with the 20th percentile of the raw data near the boundary of the colony and plotted along with the advancing speed of the swarm edge in the course of (**c**,**e**) slow spreading and (**d**,**f**) fast spreading.

As shown in Figure 3a,b, we observed the boundary region of the bacterial colony in order to investigate the interplay between the swarm colony flow pattern and the speed of the spreading edge of the colony. Observing the submicron fluorescent particles near the moving boundary of the colony at 20× magnification was very challenging. In a limited field-of-view, sometimes the beads did not move because they stuck to each other, and did not escape from the agar surface timely on occasional expansion of the colony. We therefore needed to properly locate the boundary, when both the swarming area and the stacked area were shown together. In this way, we were able to measure the instantaneous flow velocity distributions and compute the mean flow velocity and vorticity, which were the average values of the top 20% of raw data around the edge of the colony. The graphs in Figure 3c,d demonstrate the fluctuation in swarm colony flow velocity. The tendency of average flow vorticity (Figure 3e,f) was similar to that of the average flow velocity. Local swarming motility was compared to the expanding speed of the bacterial colony. In the “slow” case (Figure 3c), the speed of the swarm edge was approximately 0.2 µm/s whereas the maximum velocity of the swarm colony flow was approximately 8 µm/s. In the “fast” case (Figure 3d), the maximum speed of the swarm edge reached to 4.6 µm/s whereas the maximum flow velocity was approximately 0.5 µm/s. Overall, the velocity and vorticity of swarm colony flow were high when the spreading speed was low. Interestingly, this relationship was reversed at high spreading speed.

**Figure 4:**
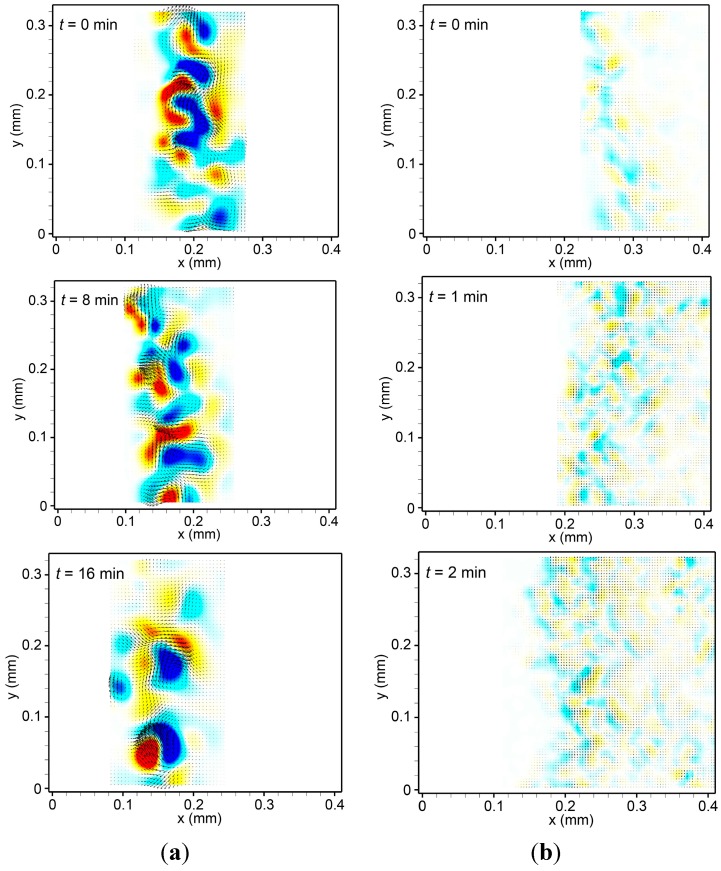
Instantaneous velocity (arrow) and vorticity (color) maps of swarm colony flow near the advancing edge of the *B. subtilis* colony at different time points in (**a**) slow spreading and (**b**) fast spreading mode. Scales of the velocity vector and vorticity are same with Figure 2.

Fauvart *et al.* [20] revealed that Marangoni flow causes spreading motion in the swarming edge by changing the gradient of surface tension in the edge, and that the surface tension gradient is reflected by the gradient of biosurfactant concentration secreted by bacterial cells. In Figure 4a,b we compare the spatial distributions of the flow velocity and vorticity at three different time points in slow and fast spreading modes, respectively. The swarm colony flow demonstrates a change of vortical pattern. The vortical pattern supports the gradient of surface tension in the entire area by generating a concentration gradient of biosurfactant. We propose that the vortical flow patterns in the swarm colony would lead to a uniform biosurfactant concentration gradient, and thus gradually increase the surface tension gradient-driven Marangoni flow, which can expand the bacterial colony. When the inner pressure is sufficient to spread, the swarm colony spreads and the flow depends on the Marangoni effect. Therefore, the average flow velocity and vorticity tend to decrease over time with high spreading speed.

## 3. Materials and Methods

### 3.1. Analysis of Fluorescent Bead Dynamics

A 20 µL of mixed solution of fluorescent bead suspension (*d* = 200 nm, λ_ex_ = 540 nm, λ_em_ = 560 nm; FluoSpheres, Invitrogen, Carlsbad, CA, USA) and 0.5% agar solution was solidified in a half part of a circular culture dish with a diameter of 11.7 mm. Bacteria-free supernatant was obtained from culture of *E. coli* or *B. subtilis* for 12 h and filtered by a sterilized syringe filter (Corning, Tewksbury, MA, USA). At 10 s after filling the other half of the culture dish with a 20 µL of each solution of distilled water, LB broth, supernatant of *E. coli*, or supernatant of *B. subtilis*, we acquired 79 images of the central area of the culture dish at 1 fps through an inverted fluorescence microscope (IX71, Olympus, Tokyo, Japan) with a 20× objective. We analyzed the images by measuring the fluorescence intensity profile at the solution using the ImageJ program (http://imagej.nih.gov/ij/) under the assumption that the intensity of the image is proportional to the concentration of the fluorescent beads. An appropriate nonlinear regression model was elaborated for analysis of the particle dynamics. Since the fluorescent beads in the agar gel diffused only to the solution across the concentration gradient, we modeled the spatiotemporal concentration, *C*(*x*, *t*), of the fluorescent beads by using Equation (1) suggested by Crank [21]:

(1) $$\frac{C\left(x,t\right)}{C_{0}}=\frac{1}{2}\left[erf\left(\frac{h-x}{2\sqrt{Dt}}\right)+erf\left(\frac{h+x}{2\sqrt{Dt}}\right)\right]$$

where *x* (µm) is the distance from a source, *t* is time (s), and *D* (µm^2^/s) is the diffusion coefficient of a solute which is assumed to be initially distributed in the region of −*h* < *x* < *h* with a uniform concentration of *C*_0_.

### 3.2. Preparation of Sample and Bacteria Culture

**Figure 5:**
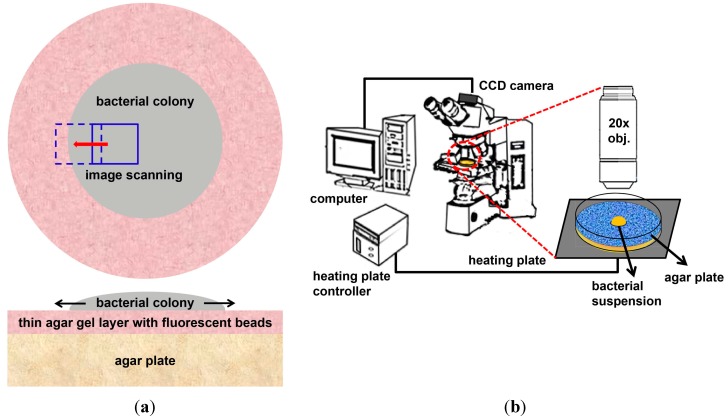
(**a**) Top and cross-sectional views of the bacterial colony growing on an agar plate, coated with a thin layer of 200 nm fluorescent microbeads; (**b**) Schematic of live cell imaging setup for visualization of fluid dynamics in swarm colony formed at the agar plate.

An agar solution of 0.5% concentration was mixed with the fluorescent microbead suspension at a ratio of 99:1 (*v*/*v*), and 200 µL of this solution was pipetted onto the 0.5% agar plate. The agar plate was rotated in a spin-coater (JSP4A, JD TECH, Seoul, Korea) at 1500 rpm for 2 s in order to deposit a thin agar gel layer containing the fluorescent microbeads on top of the agar plate. The top and cross-sectional views of the agar plate are displayed in Figure 5a. We used a glycerol stock of a *B. subtilis* (ATCC 6633) bacterial suspension and 25 g of normal nutrient (Luria-Bertani, BD, Franklin Lakes, NJ, USA), dissolved in 1 L of distilled water for incubating the bacteria at 33 °C. The bacterial culture was diluted to an optical density of 0.01, and then 1 µL of the diluted culture was inoculated into the sterilized agar gel of 0.5% (*w*/*v*) concentration.

### 3.3. Image Acquisition

A schematic diagram of the live cell imaging setup is shown in Figure 5b. The light from a mercury lamp was passed through a combinational filter and we observed the emitted fluorescence from the microparticles. We acquired both bright field and fluorescence images of *B. subtilis* cells 6–12 h after inoculation at time intervals of 150 ms using a cooled CCD camera (1600 × 1200 pixels; Sensicam, Cooke Corp., Romulus, MI, USA) installed on an upright fluorescence microscope (BX51, Olympus, Tokyo, Japan). A 20× objective enabled us to obtain the bright-field images of the swarm edge and the fluorescence images of 200 nm fluorescent beads at the same field of view. The start time for measurement was based on the moment that the movement of the fluorescent particles was clearly observed. Each image was preprocessed using the ImageJ program. The flow pattern in the *B. subtilis* bacterial colony was visualized by analyzing the images of the moving beads that were initially immobilized on top of the agar plate and subsequently diffused out into the swarm colony.

### 3.4. Data Analysis

**Figure 6:**
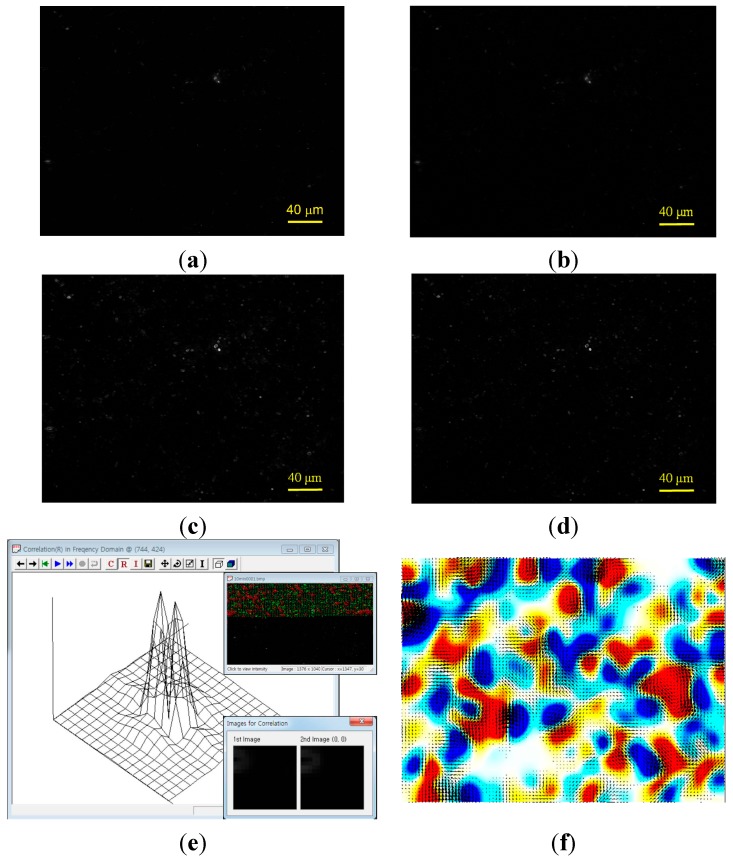
Swarm sample images of fluorescent beads undergone specific steps for image processing and analysis. (**a**) Raw image of swarm colony; (**b**) Smooth; (**c**) Find edge; (**d**) Subtract background (rolling = 10); (**e**) Particle image velocimetry analysis based on cross-correlation method; and (**f**) Velocity vector and vorticity fields.

Figure 6 shows the image processing and analysis procedure used in this study. Each raw image (Figure 6a) was pre-processed by the ImageJ program. We first removed the background noise through “Smooth” process (Figure 6b). Then “Find Edges” and “Subtract Background” processes were applied to highlight the fluorescent beads in the image (Figure 6c,d). We visualized the dynamic flow pattern near the *B. subtilis* bacterial swarm colony formed on agar gel plate. The PIV analysis based on a cross-correlation method provides the velocity field of the biosurfactant film flow in a swarm colony using a pattern-matching algorithm. When we acquire successive PIV images at time points *t*_0_ and *t*_0_ + *∆t*, small interrogation windows (*M* × *N* pixels) taken from two successive images are denoted as *IW*(*i*, *j*) and *SW*(*i*, *j*), respectively. The cross-correlation function, *R*(*p*, *q*), is defined as [22]:

(2) $$R\left(p,q\right)=\frac{\sum_{j=0}^{N-1}\sum_{i=0}^{M-1}IW\left(i-p,j-q\right)SW\left(i,j\right)}{\sqrt{\sum_{j=0}^{N-1}\sum_{i=0}^{M-1}IW^{2}\left(i,j\right)\sum_{j=0}^{N-1}\sum_{i=0}^{M-1}SW^{2}\left(i,j\right)}}$$

where *i* and *p* represent integers from 0 to *M* − 1 and likewise *j* and *q* represent integers from 0 to *N* − 1. If the maximum peak of *R*(*p*, *q*) is found at (*p*_max_, *q*_max_), the representative velocity (*u*, *v*) at the center point of the interrogation area can be determined by dividing the displacements, *p*_max_ and *q*_max_,with the time interval *∆t* and the scale factor. The algorithm was applied to the images of fluorescent particle motion induced by the collective dynamics of the bacterial cells. A custom-made program (FlowVision, Seoultech, Seoul, Korea) was used for processing instantaneous velocity vectors with an optimized set of various parameters. The interrogation size was 16 pixels. Overlapping between two adjacent interrogation areas and super-resolution analysis were not used. The computational time for calculating the cross-correlation function was dramatically reduced by applying the fast Fourier transform (FFT). A polynomial interpolation method was used to search for a peak of the cross-correlation function with a subpixel accuracy (Figure 6e). Erroneous vectors were eliminated by both range validation and peak-height validation methods.

Steager *et al.* [12] analyzed non-labelled bacterial cells in a swarm colony based on the cross-correlation method, and they could visualize the patterns formed by bacterial swarming without direct observation of fluid dynamics surrounding the bacterial swarm. We acquired images of moving submicron fluorescent beads which were coated on the agar plate initially and diffused out into the swarm colony later on. We not only obtained the information about expanding colony edge but also observed the flow pattern in growing *B. subtilis* colony. Then we measured the detailed velocity vector field of the thin biosurfactant film flow in *B. subtilis* swarm colony through PIV analysis with a cross-correlation method. The high-resolution velocity vector map was used for calculating vorticity distribution (Figure 6f). Vorticity is a measure of the local rotation of the fluid and has a positive value for a flow rotating in a counter-clockwise direction. Mean flow velocity and mean flow vorticity were calculated by averaging the 20th percentile magnitude of each parameter.

## 4. Conclusions

We revealed for the first time, based on a correlation-based PIV analysis, the detailed spatiotemporal velocity and vorticity distributions in the thin fluid layer surrounding the *B. subtilis* colony. This quantitative information would provide a new insight into the role of biosurfactant film flow in the growth and swarming dynamics of bacteria, as well as uncover a novel biophysical factor that may regulate bacterial cell physiology.
